# Cannabis Retailer Advice on Blunt, Tobacco, and Cannabis Use During Pregnancy

**DOI:** 10.1001/jamanetworkopen.2025.48373

**Published:** 2025-12-10

**Authors:** Kelly C. Young-Wolff, Monique B. Does, Rahel Negusse, Shannon N. Ogden, Joshua R. Nugent, Lynn D. Silver, Aurash J. Soroosh, Torri D. Metz

**Affiliations:** 1 Division of Research, Kaiser Permanente Northern California, Pleasanton; 2 Department of Psychiatry and Behavioral Sciences, University of California, San Francisco; 3 Prevention Policy Group Public Health Institute, Oakland, California; 4 University of Colorado School of Medicine, Aurora; 5 Denver Health and Hospital Authority, Denver, Colorado

## Abstract

**Importance:**

Pregnant individuals seeking information about the safety of prenatal cannabis use may view budtenders (individuals who work at cannabis retailers) as a trusted source of information. However, the recommendations that budtenders provide to pregnant individuals considering cannabis use are unknown.

**Objective:**

To characterize how budtenders respond to questions about the safety of blunt, tobacco, and cannabis use during pregnancy, and to evaluate whether advice varied by indication for use or by whether the retailer provided delivery.

**Design, Setting, and Participants:**

In this cross-sectional study of California cannabis retailers, mystery shoppers posed as pregnant individuals looking for advice about the safety of prenatal substance use. Telephone calls with randomly selected licensed storefront retailers were conducted from February 26, 2024, to January 28, 2025.

**Exposures:**

Mystery shoppers followed 2 versions of a script (mental health vs no mental health indications for use) with questions about the safety of prenatal blunt, tobacco, and cannabis use.

**Main Outcomes and Measures:**

The primary outcome was retailers’ responses about the safety of prenatal blunt, tobacco, and cannabis use. Secondary outcomes included product recommendations, opinions on safer vs more harmful modes of prenatal cannabis use, and whether to speak with a physician.

**Results:**

Of the 505 employees at selected retailers (ie, budtenders), 79.6% (95% CI, 74.8%-83.7%) said prenatal blunt use was unsafe, 79.2% (95% CI, 74.4%-86.4%) said prenatal tobacco use was unsafe, and 40.4% (95% CI, 35.1%-45.9%) said prenatal cannabis use was unsafe. More advised that prenatal cannabis use was safe (20.6%; 95% CI, 16.0%-26.1%) vs blunts (0.8%; 95% CI, 0.2%-2.9%) or tobacco (0.8%; 95% CI, 0.2%-2.9%) or stated that they could not give advice about the safety of prenatal cannabis (19.8%; 95% CI, 15.3%-25.2%) vs tobacco (14.3%; 95% CI, 10.5%-19.2%). Only 5.7% (95% CI, 4.0%-8.1%) mentioned store or product warnings. Budtender recommendations included low- or no-tetrahydrocannabinol cannabis products, harm reduction (eg, use less frequently), and noncannabis strategies (eg, mindfulness). Edibles were most endorsed as safe, while smoking was most endorsed as harmful. Overall, 44.0% (95% CI, 37.3%-50.8%) recommended speaking to a physician before prompting and 46.1% (95% CI, 39.4%-53.0%) after prompting. Responses were generally similar regardless of stated indication for use or delivery service availability.

**Conclusions and Relevance:**

In this cross-sectional study, most budtenders advised against prenatal blunt and tobacco use. Fewer advised against prenatal cannabis use, highlighting the need for more visible, effective warnings and mandatory budtender education covering risks of prenatal cannabis use.

## Key Points

**Question:**

How do cannabis retailer employees counsel pregnant individuals seeking information about the safety of prenatal use of blunts, tobacco, and cannabis?

**Findings:**

In this cross-sectional study of 505 California cannabis retailers, 79% of employees said prenatal blunt or tobacco use was unsafe, and 40% said prenatal cannabis use was unsafe.

**Meaning:**

These findings suggest that most cannabis retailers advised against prenatal tobacco use, but fewer did for cannabis, highlighting the need for better retailer education and consumer information.

## Teaser

This cross-sectional study evaluates how workers at cannabis retailers in California respond to questions about the safety of blunt, tobacco, and cannabis use during pregnancy.

## Introduction

While rates of prenatal cigarette use have declined,^1,2^ rates of prenatal cannabis use are increasing.^3,4,5,6^ During 2021 to 2023, the prevalence of past 30-day cannabis use among pregnant women in the US (6.5%) was similar to tobacco use (7.5%).^7^ Prenatal cannabis use is associated with adverse maternal (eg, gestational hypertension), neonatal (eg, low birthweight), and offspring neurodevelopmental outcomes,^8,9,10,11,12,13^ and medical organizations advise against prenatal cannabis use.^10,14^

Blunts (ie, hollowed out cigar wrappers filled with cannabis) are increasingly used by women,^1^ and are a common mode of cannabis administration before and during pregnancy.^1,15^ Co-use of cannabis and tobacco (vs cannabis only) is related to worse health outcomes and a lower likelihood of cannabis cessation.^16,17,18^ Compared with other ways of using cannabis, blunts have higher levels of carbon monoxide absorption and greater risk of intoxication, withdrawal, and cannabis use disorder, especially in women.^19,20,21,22,23,24,25^ Thus, blunt smoking has significant risks for pregnant individuals and their children.^26^ However, blunts may be perceived as safer than smoking cigarettes,^27^ some may not realize they contain tobacco,^28^ and flavored blunts may appeal to pregnant women who are sensitive to tobacco smell and flavor.^29^

Cannabis legalization has decreased perceptions of risk, while increasing intentions to use and providing greater acceptability and access.^30,31,32,33,34,35,36,37,38,39,40^ Pregnant individuals who use cannabis view budtenders (individuals who work at cannabis retailers) as experts on its safety and benefits during pregnancy.^41^ However, California budtenders are not required to complete job training addressing the potential harms of cannabis use, and we know little about what advice budtenders give pregnant individuals. A 2018 mystery shopper study^42^ in Colorado found that 69% of budtenders recommended cannabis to a pregnant woman for morning sickness, 36% said prenatal cannabis was safe, and 32% recommended that she speak to her clinician without prompting. Most recommendations were based on opinion rather than scientific evidence or consultation with clinicians. Another study^43^ across 5 cities in different US states found that 54.3% of retailers endorsed cannabis use for pregnancy-related nausea, and only 26.4% warned against prenatal use. Understanding how budtenders counsel pregnant individuals about cannabis, tobacco, and blunts is critical because pregnant individuals who use cannabis seek budtender advice about products, modes of use, and dosage, and their recommendations may shape risk perceptions and prenatal substance use behaviors.^41^

This mystery shopper cross-sectional study builds on prior research by characterizing actual recommendations given by budtenders in California regarding the safety of blunt, tobacco, and cannabis use during pregnancy and examining whether responses varied by stated indication for prenatal use (mental health vs none). Furthermore, because delivery availability varies across California’s storefront retailers and can reach pregnant women who avoid shopping in-person, we also tested whether budtender recommendations differed by retailers’ delivery status.

## Methods

### Setting

The Kaiser Permanente Northern California institutional review board reviewed and approved the research, and the requirement for informed consent was waived. Minor deception was necessary to obtain a realistic assessment of what cannabis retailers recommend for pregnant individuals. All retailers in California were informed of the results upon publication of the study. We followed the Strengthening the Reporting of Observational Studies in Epidemiology (STROBE) reporting guideline for cross-sectional studies.

### Procedures

This cross-sectional population-based survey study used a mystery caller approach following methods similar to Dickson et al.^42^ The study team developed 2 scripts to examine recommendations from California budtenders around prenatal use of blunts, tobacco, and cannabis based on the literature and study team expertise (Box and Table 1). The script queried about the safety of prenatal blunt use; if a budtender stated that blunts were not safe during pregnancy, the caller further clarified whether it was the tobacco, cannabis, or both that was unsafe. The script solicited recommendations for what type of cannabis to use during pregnancy, which modes are safer or more harmful during pregnancy, whether one should talk to their health care practitioner about prenatal cannabis use, and whether the retailer provides delivery services (ie, “do you deliver?”). Additionally, mystery shoppers coded whether the retailers noted the source of the recommendation offered (eg, personal experience, research studies, or warning labels). To test whether budtender responses were different when there was a mental health reason for prenatal cannabis use, one script emphasized that the caller was using blunts for mental health reasons and the other did not. We hypothesized that budtenders would be more supportive of prenatal cannabis use for mental health vs non–mental health reasons.

Box. **Phone Scripts for Anonymous Survey of Cannabis Retailers^a^**

**Script 1: Mental Health Reasons for Use**

Question 1: “Hi, I usually smoke blunts when I’m feeling anxious or depressed but now I’m pregnant. I was wondering if it’s safe to use blunts?”
If unsafe/don’t know/can’t provide medical advice: “Is it the tobacco or cannabis that is not safe?” (if not mentioned).
If safe: Record any reasons given for why it is safe.
Question 2: “Is there anything else you would recommend for anxiety while I am pregnant?” (if not mentioned).
Record any products or recommendations.
Question 3: “What’s the safest way to use while I am pregnant? Like smoking or vaping or using edibles?” (if not mentioned).
Record any reasons given for why any given mode is safer than others, and code if specific modes are reported safe or unsafe.
Question 4: “Do you think it’s worth talking to my doctor about using weed while I’m pregnant?” (if not mentioned).
Question 5: “Thank you for answering my questions. It has been super helpful. One last question, do you deliver?”
Document whether the respondent states if the harms are more related to the baby or the mom.
Document whether the respondent cites a source of information (personal opinion, referenced research, online sources, referenced retailer policy, posters or warnings in dispensary, or did not specify).

**Script 2: No Stated Reason for Use**

Question 1: “Hi, I usually smoke blunts but now I’m pregnant. I was wondering if it’s safe to use blunts?”

If unsafe/don’t know/can’t provide medical advice: “Is it the tobacco or cannabis that is not safe?” (if not mentioned).
If safe: Record any reasons given for why it is safe.

Question 2: “Is there anything else you would recommend while I am pregnant?” (if not mentioned).

Record any products or recommendations.

Question 3: “What’s the safest way to use while I am pregnant? Like smoking or vaping or using edibles?” (if not mentioned).

Record any reasons given for why any given mode is safer than others, and code if specific modes are reported safe or unsafe.

Question 4: “Do you think it’s worth talking to my doctor about using weed while I’m pregnant?” (if not mentioned).
Question 5: “Thank you for answering my questions. It has been super helpful. One last question, do you deliver?”
Question 6: Document whether the respondent states if the harms are more related to the baby or the mom.
Document whether the respondent cites a source of information (personal opinion, referenced research, online sources, referenced retailer policy, posters or warnings in dispensary, or did not specify).

^a^
The wording of questions 1 and 2 are slightly different; all other questions are identical across scripts. Table 1 footnotes contain additional information.

**Table 1.** Cannabis Retailer Responses to Questions About Blunt, Tobacco, and Cannabis Use During Pregnancy, by Script

Characteristic	Overall, No. (%) [95% CI] (N = 505)	Script type		*P* value
		Mental health indication script, No. (%) [95% CI] (n = 243)	Non–mental health indication script, No. (%) [95% CI] (n = 262)	
Prenatal blunt use^a^				
Safe	4 (0.8) [0.2-2.9]	1 (0.4) [0.0-3.9]	3 (1.2) [0.3-4.9]	.40
Not safe	402 (79.6) [74.2-84.2]	188 (77.4) [69.1-84.0]	214 (81.7) [74.1-87.4]	
Unsure	15 (3.0) [1.5-5.9]	9 (3.7) [1.5-8.8]	6 (2.3) [0.8-6.6]	
Cannot give medical advice	84 (16.6) [12.5-21.8]	45 (18.5) [12.6-26.4]	39 (14.9) [9.8-22.1]	
Prenatal tobacco use^a^				
Safe	4 (0.8) [0.2-2.9]	1 (0.4) [0.0-3.9]	3 (1.2) [0.3-4.9]	.04
Not safe	400 (79.2) [73.7-83.8]	188 (77.4) [69.1-84.0]	212 (80.9) [73.3-86.8]	
Unsure	29 (5.7) [3.5-9.4]	10 (4.1) [1.8-9.3]	19 (7.3) [3.9-13.1]	
Cannot give medical advice	72 (14.3) [10.5-19.2]	44 (18.1) [12.2-26.0]	28 (10.7) [6.5-17.2]	
Prenatal cannabis use^a^				
Safe	104 (20.6) [16.0-26.1]	52 (21.4) [15.0-29.6]	52 (19.9) [13.9-27.6]	<.001
Not safe	204 (40.4) [34.5-46.6]	97 (39.9) [31.6-48.9]	107 (40.8) [32.7-49.5]	
Unsure	97 (19.2) [14.8-24.6]	28 (11.5) [7.0-18.5]	69 (26.3) [19.5-34.6]	
Cannot give medical advice	100 (19.8) [15.3-25.2]	66 (27.2) [20.0-35.8]	34 (13.0) [8.2-19.9]	
Recommendations for prenatal use^b^				
Low or no THC^c^	185 (36.6) [32.6-40.9]	119 (49.0) [42.8-55.2]	66 (25.2) [20.3-30.8]	<.001
Other harm reduction strategies^d^	111 (22.0) [18.6-25.8]	57 (23.5) [18.6-29.2]	54 (20.6) [16.2-25.9]	.44
Noncannabis (eg, mindfulness)^e^	31 (6.1) [4.4-8.6]	13 (5.4) [3.2-8.9]	18 (6.9) [4.4-10.6]	.48
No recommendations	239 (47.3) [43.0-51.7]	90 (37.0) [31.2-43.3]	149 (56.9) [50.8-62.7]	<.001
Are certain modes safer than others^a^				
Certain modes safer	211 (41.8) [35.2-48.6]	107 (44.0) [34.6-53.9]	104 (39.7) [30.9-49.2]	.51
No difference in safety by mode	35 (6.9) [4.2-11.3]	16 (6.6) [3.1-13.3]	19 (7.3) [3.7-13.8]	
No mode is safe	70 (13.9) [9.8-19.3]	30 (12.4) [7.2-20.3]	40 (15.3) [9.7-23.3]	
Do not know	121 (24.0) [18.6-30.3]	59 (24.3) [16.9-33.6]	62 (23.7) [16.6-32.6]	
Did not ask	68 (13.5) [9.5-18.8]	31 (12.8) [7.5-20.8]	37 (14.1) [8.8-22.0]	
Modes that are safer^b^				
Smoking^f^	28 (5.5) [3.9-7.9]	16 (6.6) [4.1-10.4]	12 (4.6) [2.6-7.8]	.33
Vaping^g^	11 (2.2) [1.2-3.9]	6 (2.5) [1.1-5.3]	5 (1.9) [0.8-4.4]	.67
Dabbing^h^	4 (0.8) [0.3-2.0]	2 (0.8) [0.2-3.0]	2 (0.8) [0.2-2.7]	>.99
Edibles^i^	160 (31.7) [27.8-35.9]	87 (35.8) [30.0-42.0]	73 (27.9) [22.8-33.6]	.06
Sublingual^j^	5 (1.0) [0.4-2.3]	2 (0.8) [0.2-3.0]	3 (1.2) [0.4-3.3]	>.99
Topical^k^	26 (5.2) [3.5-7.4]	12 (4.9) [2.9-8.4]	14 (5.3) [3.2-8.8]	.84
Modes that are less safe^b^				
Smoking^f^	128 (25.4) [21.8-29.3]	57 (23.5) [18.6-29.2]	71 (27.1) [22.1-32.8]	.35
Vaping^g^	31 (6.1) [4.4-8.6]	11 (4.5) [2.6-7.9]	20 (7.6) [5.0-11.5]	.15
Dabbing^h^	5 (1.0) [0.4-2.3]	1 (0.4) [0.0-2.3]	4 (1.5) [0.1-3.9]	.37
Edibles^i^	24 (4.8) [3.2-7.0]	8 (3.3) [1.7-6.4]	16 (6.1) [3.8-9.7]	.14
Sublingual^j^	1 (0.2) [0.0-1.1]	0 (0.0) [0.0-1.6]	1 (0.4) [0.1-2.1]	>.99
Topical^k^	0	0	0	NA
Worth talking to physician^a^				
Yes (already suggested)	222 (44.0) [37.3-50.8]	116 (47.7) [38.1-57.5]	106 (40.5) [31.6-50.0]	.51
Yes (when prompted)	233 (46.1) [39.4-53.0]	106 (43.6) [34.2-53.5]	127 (48.5) [39.2-57.9]	
No	11 (2.2) [0.9-5.2]	4 (1.7) [0.4-6.5]	7 (2.7) [0.9-7.8]	
I do not know	29 (5.7) [3.3-9.8]	12 (4.9) [2.1-11.2]	17 (6.5) [3.2-12.9]	
Did not ask	10 (2.0) [0.8-5.0]	5 (2.1) [0.6-7.2]	5 (1.9) [0.5-6.7]	
Source of information^b^^,^^l^				
No source	228 (45.2) [40.9-49.5]	120 (49.4) [43.2-55.6]	108 (41.2) [35.4-47.3]	.07
Personal experience or opinion	180 (35.6) [31.6-39.9]	80 (32.9) [27.3-39.1]	100 (38.2) [32.5-44.2]	.22
General knowledge	46 (9.1) [6.9-11.9]	20 (8.2) [5.4-12.4]	26 (9.9) [6.9-14.1]	.51
Published research	35 (6.9) [5.0-9.5]	10 (4.1) [2.3-7.4]	25 (9.5) [6.6-13.7]	.02
Online sources (eg, search engines)	40 (7.9) [5.9-10.6]	18 (7.4) [4.7-11.4]	22 (8.4) [5.6-12.4]	.68
Product or retailer warnings	29 (5.7) [4.0-8.1]	14 (5.8) [3.5-9.4]	15 (5.7) [3.5-9.2]	.99
Lack of reliable information	22 (4.4) [2.9-6.5]	8 (3.3) [1.7-6.4]	14 (5.3) [3.2-8.8]	.26
Delivery services available^a^^,^^m^				
Yes	166 (32.9) [28.9-37.1]	89 (36.6) [30.8-42.9]	77 (29.4) [24.2-35.2]	.08
No	339 (67.1) [62.9-71.1]	154 (63.4) [57.2-69.2]	185 (70.6) [64.8-75.8]	

Abbreviations: NA, not applicable; THC, tetrahydrocannabinol.

^a^ Categories are mutually exclusive.

^b^ Categories are not mutually exclusive.

^c^ Products with no THC (eg, products with only non-THC cannabinoids such as cannabidiol, cannabinol, etc.) and low THC products (eg, products noted as containing a THC:non-THC ratio or a lower-than-average THC content) were combined into 1 category called low or no-THC.

^d^ Harm reduction strategies include suggestions to use alternate (safer) methods of consuming that may still contain cannabis (eg, using clean methods like a bong or pipe, switching to hemp blunt wraps, avoiding chemicals and pesticides) and suggestions to simply consume less cannabis.

^e^ Noncannabis recommendations include supplements and lifestyle modifications such as diet, mindfulness, yoga, and meditation.

^f^ Smoking includes the use of joints, bongs, pipes, and blunts.

^g^ Vaping is use of an electronic device that heats liquid or dry material to produce an aerosol or vapor (eg, vape pens or desktop vaporizers).

^h^ Dabbing is the use of cannabis concentrates (eg, shatter or wax).

^i^ Edibles includes gummies, tablets, capsules, tinctures, drinks, foods, and other orally ingested products.

^j^ Sublingual are tablets or strips that dissolve under the tongue.

^k^ Topicals are cannabis products applied directly to and absorbed through the skin (eg, lotion, ointments, or bath bombs).

^l^ Source of information was recorded after the call based on the caller’s assessment of the information provided. If the budtender stated an obvious personal opinion (eg, “I believe…,” or “In my opinion…”), the response was coded as personal opinion. If the budtender cited specific research studies or specific websites, the response was coded as published research or online sources, respectively. If the budtender referenced specific retailer policies (eg, this store prohibits giving medical advice) or warnings on products or posters displayed in the dispensary, responses were coded accordingly. If responses reflected commonly held beliefs, (eg, “most people say” or “it is generally not recommended”) without citing another source, responses were coded as general knowledge.

^m^ Caller was asked at the end of the call if the retailer provided delivery services (yes responses included limited delivery service; ie, particular times of day and geographic restrictions).

Three mystery shoppers from the study team (K.Y.W., M.B.D., and R.N.) conducted the calls using REDCap, a secure web application. Calls were conducted from February 26, 2024, to January 28, 2025, and a maximum of 3 calls were made to each retailer. Due to California laws, calls were not recorded, but mystery shoppers took near verbatim notes (directly into REDCap) and coded responses in REDCap using predetermined categories (see Table 1). Callers did not request or record any identifying information from budtenders. Scripts were developed iteratively and tested across 22 Oregon retailers before they were finalized. All callers completed standardized training, including practice calls and role-playing to ensure consistency of delivery and documentation. Callers met regularly to review notes, compare budtender responses, and discuss codes. The lead caller (M.B.D.) reviewed notes and codes for all calls for completion, accuracy, and consistency. All coding discrepancies were resolved through discussion and consensus.

### Sample

The target population consisted of all 1167 storefront retailers with a medical and adult-use cannabis license in California as of November 17, 2023, using license data from the California Department of Cannabis Control (DCC) website. To achieve feasible call volume, we randomly selected 800 storefront retailers and assigned them to 1 of the 2 scripts using block randomization by county and then randomized to the 3 mystery callers. Two retailers subsequently identified as duplicates at the same physical address were removed, yielding a final randomly selected sample of 798 retailers. Retailer telephone numbers were obtained by the callers through an online search of the retailer’s name cross-checked against the corresponding address in the DCC list, as the telephone number listed by DCC was typically for the retailer owner rather than the retailer.

### Statistical Analysis

The primary outcome was the percentage of retailers who advised that blunt use, tobacco use, and cannabis use were unsafe during pregnancy. Secondary outcomes included recommendations given, safer or more harmful modes of cannabis use, and the percentage who recommended the caller speak with their health care practitioner (with or without prompting). Responses from the mental health vs non–mental health scripts and delivery services vs nondelivery services retailers (based on budtender’s responses) were compared using exact Pearson χ^2^ tests using SAS version 9.4 (SAS Institute); 95% CIs were computed using the Quesenberry and Hurst method. A 2-sided *P* < .05 was considered statistically significant. Analyses were conducted from March to June 2025. Representative quotations from retailers were selected to supplement the quantitative findings.

## Results

Of the 798 retailers to whom calls were attempted, 293 (36.7%) were not completed (213 [26.7%] had no working number, 76 [9.5%] had no answer, 4 [0.5%] refused or were ineligible). Valid calls were achieved for 505 retailers (63.3%; 243 mental health, 262 non–mental health scripts) (eFigure in Supplement 1); the Figure shows the distribution of retailers with valid calls across California.

**Figure.**
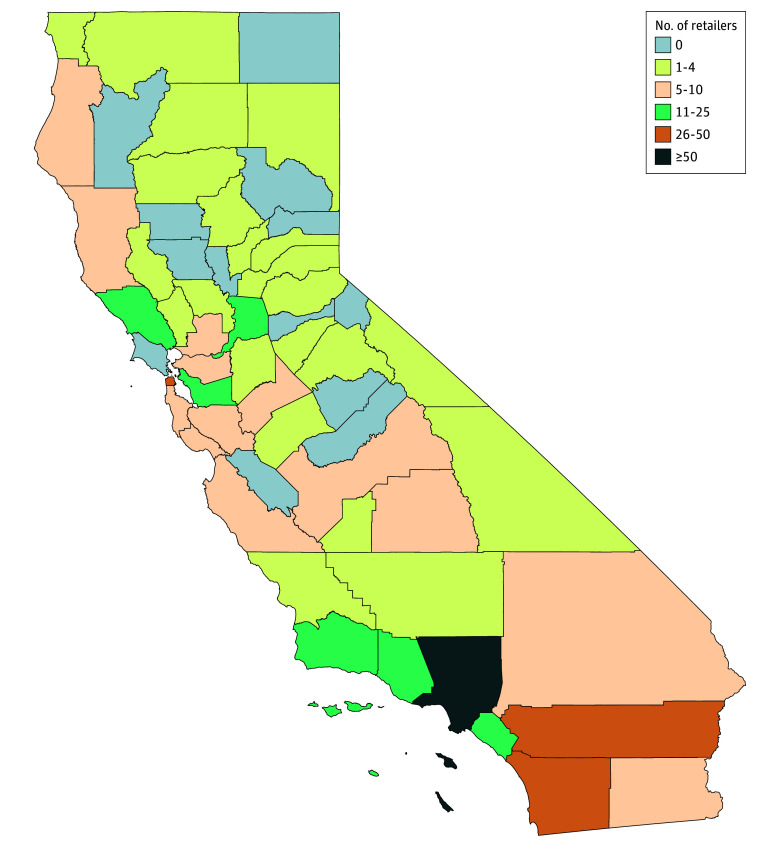
Number of Retailers Per County in Sample

Overall, 0.8% (95% CI, 0.2%-2.9%) of budtenders advised that prenatal blunt use was safe, 79.6% (95% CI, 74.2%-84.2%) advised that it was unsafe, 3.0% (95% CI, 1.5%-5.9%) said they were unsure, and 16.6% (95% CI, 12.5%-21.8%) said they could not provide medical recommendations (eg, “I am not a doctor and cannot advise you on this”) (Table 1). Similarly, when probed specifically about safety of tobacco and cannabis (2 main components of blunts), 0.8% (95% CI, 0.2%-2.9%) advised that prenatal tobacco use was safe, 79.2% (95% CI, 73.7%-83.8%) advised it was unsafe, 5.7% (95% CI, 3.5%-9.4%) were unsure, and 14.3% (95% CI, 10.5%-19.2%) said they could not provide medical advice. In contrast, when probed, 20.6% (95% CI, 16.0%-26.1%) advised that prenatal cannabis use was safe, 40.4% (95% CI, 34.5%-46.6%) advised that it was unsafe, 19.2% (95% CI, 14.8%-24.6%) were unsure, and 19.8% (95% CI, 15.3%-25.2%) said they could not provide medical advice. When addressing safety concerns, 89 (17.6%; 95%CI, 13.7%-22.1%) mentioned potential harms for the baby and 12 (2.4%; 95%CI, 1.2%-4.7%) mentioned potential harms for mother and baby. Table 2 includes representative quotes from budtenders in response to phone script questions, highlighting the inconsistent advice given. Overall, 36.6% (95% CI, 32.6%-40.9%) recommended low- or no-tetrahydrocannabinol (THC) cannabis products containing other cannabinoids (eg, cannabidiol), 22.0% (95% CI, 18.6%-25.8%) recommended other harm reduction strategies (eg, consuming less frequently), and 6.1% (95% CI, 4.4%-8.6%) recommended noncannabis strategies (eg, mindfulness).

**Table 2.** Representative Quotes From Budtenders in Response to Phone Scrip Questions

Topic	Representive quote
Prenatal blunt use	
Safe	“I think you will be fine. I smoked blunts and joints while I was pregnant and my kids are fine.”
Not safe	“I would not recommend blunts because the tobacco isn't safe. Joints and out of pipe is fine and safe.” “Absolutely no! Terrible for your baby. Can cause mental retardation and birth defects. You must stop cold turkey now.”
Unsure	“I am not sure, it is really up to you.”
Cannot give medical advice	“We're not able to answer that question. Your best bet is to ask your doctor.” “Not being a medical professional, I don't like to give out information, especially when you're pregnant because I don't want to give you wrong information.”
Prenatal tobacco use	
Safe	“I think you will be fine.”
Not safe	“Tobacco products are not safe. There are warning labels on them.” “Definitely tobacco is not safe. Switch to hemp wraps.”
Unsure	“Probably both cannabis and tobacco are unsafe, but I don’t know for sure.”
Cannot give medical advice	“I’d ask your doctor about this. Not to be rude, but we can’t tell you for sure that something is safe.”
Prenatal cannabis use	
Safe	“Joints and out of a pipe is fine and safe.” “They have done research with cannabis, and it doesn’t cause harm.” “Marijuana is safe. I smoked my whole pregnancy and my daughter is fine, she is a-ok. It helped me to eat things.” “Cannabis is okay and safe before five months.” “As long as you stay away from the tobacco. A lot of people say that cannabis is harmful, but that’s a stereotype.”
Not safe	“Cannabis has a label that says not safe in pregnancy.” “From what I’ve read, everything you consume when pregnant your baby will also consume, and that it might affect them well into their twenties.”
Unsure	“Marijuana use, I’m not sure. Best thing is to ask your doctor.” “New research says marijuana may not be bad, but we don’t know.”
Cannot give medical advice	“Scientific-wise, do your own research. I can’t give medical advice.”
Recommendations for prenatal use (not mutually exclusive)	
Low or no THC	“Low THC, high CBD are safer.” “CBD tinctures would be good.” “Maybe CBN only.” “Only CBD products with no THC.” “High CBD products are the way to go, CBD is natural, you pass it to your baby through breast milk. THC is dangerous for the baby, how bad I can't really say.”
Other cannabis-related harm reduction strategies	“Bath bombs would be a good option.” “Cut back, don’t smoke all day.” “Avoid high concentration products, infused products.” “We have lotions you can try.” “Use natural hemp wraps instead of blunts.” “If you use during pregnancy, definitely get it from a dispensary.” “Smoking ‘clean’ is best from a bong or pipe. Studies show that what is bad is what you smoke out of not what you are smoking.” “Don’t use during the third trimester.” “Safest in third trimester, in moderation.” “Use the minimal amount and consult with your doctor.” “Use clean methods like bong, pipe, and avoid papers.”
Noncannabis (eg, mindfulness)	“Eat healthy, take care of yourself.” “Lion’s mane mushrooms for relaxation and anxiety, brain health.” “Use black pepper to help with cravings. Lavender, sea moss, and chamomile for stress.” “Spend time with loved ones, relaxation, massage, exercise, eat well.” “Try chewing on ice cubes, can help with oral fixation and hydrate you.” “Bananas and vitamin B6 are good for morning sickness.” “Kava root.” “Herbal teas. Ask your doctor what you can do instead of cannabis. There are lots of good options that don't require smoking or using drugs.” “Meditation, breathing techniques. I have bad anxiety and have learned to cope using breathing.” “Lavender essential oil and chamomile tea. My mom did acupuncture when she was pregnant with me and it helped her with her anxiety. Peppermint oil is helpful for nausea. No alcohol. If you are petite, make sure to take iron.” “Again, talk to doctor, but maybe try magnesium supplements to help you relax.”
No recommendations	“Can’t recommend anything while pregnant, not even CBD.” “Talk to your doctor, I really can’t help you.” “I can’t give medical advice.” “Stop completely. I can’t recommend anything.” “I would say CBD but that might also be bad. None of us are doctors so we can't give medical advice. I would say you should ask your doctor to help you address your anxiety in other ways.”
Are certain modes safer than others	
Certain modes safer	See responses in next section under safer or less safe modes.
No difference in safety by mode	“However you want to use it is good.” “Doesn’t matter. Just reduce the amount you are using it.” “I think all modes are about the same. They have pros and cons.”
No mode is safe	“Any THC is bad for baby. In California, they test you at birth for drugs and if positive, they will call child protective services and take your baby away.” “No way of using cannabis is safe.” “Don’t do anything. Everything you put in your mouth the baby feels. I would quit cold turkey. You could think, ‘Does my baby want to be high today?’…If you were my girl I would beg you not to do any of this, at least until the baby is born. I’m not shaming. Just sit back and think about what do you want for your child.” “THC will get to your baby no matter how you do it so best to stop.”
Do not know	“I don’t know honestly. Ask your doctor.” “There really hasn’t been enough research on this, some doctors will admit that.”
Modes that are safer (not mutually exclusive)	
Smoking	“Compared to vaping, anything you smoke will be less harmful because it goes right to the brain and impacts the baby less.” “Use from a pipe. That’s safer than edibles or vaping because edibles go straight to the baby.”
Vaping	“I'm not sure, vaping might be safest.” “Vaping and edibles are safest, need to avoid soot build up in body.”
Dabbing	“Pot does have herb so it's carcinogenic. I would switch to a cartridge with a dab pen as clean as possible. Liquid diamond cart is super pure. Plant is stripped of THC, filtered, and then vaporized. Live resin is better. You could do an electric dab rig or anything that is a concentrate.”
Edibles	“Edibles might be safer than smoking.” “Go as natural as possible. Capsule or tincture would be the best option.” “Edibles are safer. Talk to your doctor. My doctor told me to do edibles.” “Pregnant people usually use edibles because of fewer carcinogens.” “A lot of people switch to edibles and beverages.” “Fast-acting edibles are a good choice.”
Sublingual	“There are drops that you use under your tongue. The one I think might be good is made by Unique Karma, you should look them up and do some research.”
Topical	“Bath bombs and topicals.” “My baby mama used. Honestly, it would be better to use a topical. Don’t want to get in trouble with child protective services.” “Body creams without THC.” “Topicals, patches. Those won’t go directly to the baby.” “I really can't say, we have balm, but that wouldn't help you with anxiety, and pretty much anything else that you take into your body will be in the bloodstream and enter the umbilical cord. You really should speak to your doctor about this.”
Modes that are less safe (not mutually exclusive)	
Smoking	“Smoking is not as safe.” “Don’t recommend smoking anything.” “Smoking is just all around bad.” “Mode matters, especially during pregnancy. Definitely don't smoke. You could try topicals.”
Vaping	“Stay away from vapes!” “Vaping is worse in my personal opinion. A lot more has been done to it to get the effects. Eating might be equal to or better than smoking, but I can't recommend anything.” “My doctor said edibles are the safest, no vaping, too new with side effects.”
Dabbing	“Stick to papers, joints, and smoking. Don't do dabs.”
Edibles	“Edibles might be more harmful because they go to your saliva glands and hit you more directly.” “Edibles go into your digestive system and anything you eat the baby eats.” “Edibles will go straight to the baby.” “Edibles are worse than smoking as liver absorbs seven times as much THC.” “Definitely not edibles, anything you eat goes right to the baby. Inhaling smoke doesn't affect baby.” “I think edibles are too potent and have too many additives. If you need to use, stick with joints or bongs.”
Worth talking to doctor	
Yes (already suggested)	“I am not able to give medical advice. You should ask your doctor.” “Most statistics say it’s not safe for the baby’s development. But everyone is different, so you should talk to your doctor. There might be medical reasons why it is okay to use.” “Definitely no tobacco. I can't really say about cannabis. It is personal decision. I am pregnant too, and choosing to stop. But you should talk to your doctor.” “I'm sorry but I don't think I'm qualified to say, you need to ask a medical person that.” “We have products for anxiety, but not ones that I can recommend while you're pregnant, maybe after you speak to your doctor they can make a recommendation for you.” “We won't deny you, but you need to talk to your doctor.” “This is a doctor question, don't mean to be rude or nothing, but I couldn't tell you what's safe for your body. There are a lot of factors and the only person who can give you that information is a doctor.”
Yes (when prompted)	“Absolutely. Talk to your doctor. They caution us to stay away from these conversations.” “Definitely talk to your doctor. We are not medical experts.” “Yes, talk to your doctor. 110% yes. I’m not a doctor. I’m telling you about experiences from other women.” “Sure. Talk to him. And see if he has other things he recommends.” “Better to be safe and ask your doctor.” “Yes, absolutely. And if your doctor says that THC is fine, we can then recommend some products that might work.” “Yes, they are more knowledgeable and understanding now.” “Yes, definitely talk to your doctor. There is a lot of misinformation out there and they can help sort it out.” “I think talking to your doctor is an excellent idea. They are the experts.”
No	“No. They will just tell you not to use anything.” “Hmmm…It’s changed so much in the last few years with legalization. I wouldn’t mention it.” “I’ve heard too many horror stories and child protective services getting involved.”
I do not know	“I don’t know. Most doctors don’t give a shit. They’ll just give you pills. I’m not a doctor.” “It depends on how chill your doctor is.” “You can, but I can’t say what would happen. I don’t know.”
Source of information (not mutually exclusive)	
Personal experience or opinion	“I used with my daughter, and she is perfectly healthy.” “You'll want to ask your doctor. I personally have done the same. I used rarely while I was pregnant and did my own research on google. My doctor was adamant not to do it in first 2 to 6 months because that is when the brain is developing but after that it is okay. I also used it for throwing up or to help me be hungrier.” “Cannabis is fine. My cousin's baby came out as a genius, no difficulties with birth. Stop a month before so it's not in your blood.” “My mom smoked weed during pregnancy with us and we all turned out okay.”
General knowledge	“Yeah, there are a lot of debates on this, it's kind of all up in the air. Some people say it's fine as long as you stop at a certain point in pregnancy." “In general, based on what I have been told, you shouldn't smoke anything while you are pregnant.” “They say not to. Smoking is not good.”
Published research	“We do not recommend any type of cannabis during pregnancy, studies have shown bad things happen when you do that.” “Research says using cannabis is safe while you are pregnant.”
Online sources (search engines)	“Go online to do your own research. Go to Leafly.” “Smoking is not good while pregnant. Go online and do your own research about what is best during pregnancy. Lots of opinions and information out there!”
Retailer policy	“For legal reasons, it is our policy that we are not allowed to answer. We can't give medical advice about pregnancy.” "I'm sorry but as a business we can't answer questions like that, sorry."
Product or retailer warnings	“Generally, most people try to stay away from smoking because California warnings say it can cause reproductive harm.” “It's advised against, warnings on all products, we do have people who are pregnant and we sell to them, but we can't confirm it’s safe.” “All of our products in the store have a warning label that says not to use during pregnancy.” “We have printouts in the store that come from the San Francisco Department of Public Health, and it says they discourage smoking or using cannabis while pregnant.”
Lack of reliable information	“It is 50/50; some people say you shouldn't but I know many people who have smoked throughout pregnancy and kids are OK. Can’t really say yes or no, you should really talk to your doctor. I definitely think you should stop tobacco, but I don’t think cannabis is harmful to baby.” “Yeah, there are a lot of debates on this. It's kinda all up in the air. Some people say it's fine as long as you stop at a certain point in pregnancy. It’s really up to you and, you know, I really can't say for sure.”

Abbreviations: CBD, cannabidiol; CBN, cannabinol; THC, tetrahydrocannabinol.

Overall, 41.8% (95% CI, 35.2%-48.6%) endorsed that some modes of prenatal use are safer or more harmful (including those who originally said use is not safe, but went on to suggest something; eg, “No use is best, but if need to use, then edibles are probably safest”), 24.0% (95% CI, 18.6%-30.3%) did not know, 6.9% (95% CI, 4.2%-11.3%) said safety or risk does not vary by mode, and 13.9% (95% CI, 9.8%-19.3%) said no modes are safe. Edibles were the most endorsed safer mode (160 respondents [31.7%]), followed by smoking (28 respondents [5.5%]), topicals (26 respondents [5.2%]), vaping (11 respondents [2.2%]), sublingual (5 respondents [1.0%]), and dabbing (4 respondents [0.8%]). Smoking was most endorsed as a less safe mode (128 respondents [25.4%]) followed by vaping (31 respondents [6.1%]), edibles (24 respondents [4.8%]), dabbing (5 respondents [1.0%]), and sublingual ( respondent [0.2%]). This question was not asked when the budtender had clearly advised against any prenatal cannabis use and the caller perceived this question might cause discomfort (68 respondents).

During the call, 222 budtenders (44.0%) recommended speaking to a physician without prompting, and 233 (46.1%) recommended it when asked if the caller should talk to her physician about prenatal cannabis use (455 overall [90.1%]). While almost half did not spontaneously share their source of information for their recommendation (228 respondents [45.2%]), personal experience was most common (180 respondents [35.6%]), followed by general knowledge (eg, “most people say”; 46 respondents [9.1%]), online sources (eg, search engines, social media, and blogs; 40 respondents [7.9%]), and published research (35 respondents [6.9%]). Only 29 [5.7%] mentioned product or store warnings.

Responses were generally similar for the mental and non–mental health scripts, with a few key differences. Budtenders who received the mental health script were less likely to endorse prenatal tobacco use as safe (1 respondent [0.4%] vs 3 [1.2%]) and more likely to say they could not say whether tobacco is safe (44 respondents [18.1%] vs 28 [10.7%]) (Table 1). They were also more likely to report they could not say whether prenatal cannabis is safe (66 respondents [27.2%] vs 34 [13.0%]), but less likely to say they were unsure (28 respondents [11.5%] vs 69 [26.3%]). Budtenders with the mental health script were also more likely to recommend low or no THC products (119 respondents [49.0%] vs 66 [25.2%]), less likely to have no recommendations (90 respondents [37.0%] vs 149 [56.9%]), and less likely to cite published research as a source of their information (10 respondents [4.1%] vs 25 [9.5%]). Responses were similar among retailers with and without delivery services (eTable in Supplement 1).

All callers noted empathy among budtenders (eg, acknowledging that prenatal use is a personal decision and can be a difficult choice if cannabis is helping with mental and physical health). For example, one budtender stated: “I recognize how complicated a question like this is. If you need any help at all and want to come in, I’m happy to work personally with you.”

## Discussion

In this cross-sectional mystery caller study of 505 randomly selected storefront cannabis retailers across California, we characterized budtender recommendations around prenatal blunt, tobacco, and cannabis use. Less than 1% said that prenatal blunt or tobacco use was safe, while 20% said that prenatal cannabis use was safe. A lower percentage said they were unsure about the safety of prenatal blunt or tobacco use vs prenatal cannabis use, only 40% advised that prenatal cannabis use was unsafe, and more stated that they could not give advice about the safety of prenatal cannabis vs blunt or tobacco use. Overall, edibles were endorsed as a safer mode of prenatal cannabis use and smoking was endorsed as a more harmful mode of use. Responses were inconsistent across retailers, with some indicating prenatal smoking was safest. This discrepancy highlights the lack of research and education on the relative harms of different modes of prenatal cannabis use.

Despite required budtender training in California, pregnant individuals who used cannabis report viewing them as experts on the safety and benefits of prenatal cannabis use and view them as a trusted source for accurate advice about products.^41^ Our findings complement results from a mystery caller study in Colorado that found that 69% of budtenders recommended cannabis to a pregnant woman for morning sickness, 36% said prenatal cannabis was safe, and only 32% recommended that she speak to her clinician without prompting (80% ultimately recommended discussion following a direct query). Results are also consistent with a recent study across 5 cities in different US states that found 54.3% of retailers endorsed cannabis use for pregnancy-related nausea, and only 26.4% warned against prenatal use.

Recommendations from budtenders included low or no THC cannabis products, other harm-reduction related advice (eg, use less frequently, avoid additives and pesticides, and use clean methods like flower from a bong or pipe), and health strategies not involving cannabis (eg, mindfulness or herbal tea). Overall, 90.1% ultimately recommended speaking with a physician, with about half (44.0%) recommending this without prompting. Notably, most budtenders conveyed warmth and empathy and acknowledged the complex factors that go into decision-making about prenatal cannabis use even as they often provided incorrect guidance.

Our study is novel in its examination of whether retailer responses varied based on callers’ reasons for prenatal cannabis use. Callers who reported using cannabis for mental health indications received more cautious budtender recommendations, including advice to use low or no THC products. Budtenders may be more careful about making recommendations when pregnant customers use cannabis for mental health reasons vs recreationally. Furthermore, budtenders may believe THC is the only harmful cannabinoid or consider other cannabinoids as only having medicinal properties. Additional studies are needed to further investigate the factors underlying differences in advice.

Existing research not specific to pregnancy shows that budtenders self-report lack of training related to cannabis therapeutics (with training focused more on sales/profit vs health promotion) and would like high-quality training opportunities.^44^ Budtenders report making recommendations to cannabis buyers based on other customer experiences and their own experiences.^45^ A study in Washington found that budtenders were amenable to handing out or posting information from the state related to risky use behaviors (eg, use while driving or during pregnancy).^46^ In this study, most budtenders who mentioned their source of information reported that their advice was based on personal experience or opinion. While the exact factors underlying the inconsistent recommendations around the safety of prenatal cannabis use are unknown, it is possible that knowledge gaps, misinformation, personal beliefs, the influence of the cannabis industry, or desire to sell products may be contributing factors. Budtenders may not feel confident answering questions related to the safety of prenatal cannabis use but may feel pressured by customers’ expectations or the desire to sell cannabis. Understanding the mechanisms underlying inconsistent recommendations can lead to the development of more informed training, communication, and regulatory strategy interventions.

Notably, only 5.7% of retailers mentioned on-package or in-retailer warnings about prenatal cannabis use, highlighting the need for improved awareness and visibility of warnings. Results suggest that the required health warnings on cannabis product packaging in California, which can be in size 6 font, including one stating, “CANNABIS USE WHILE PREGNANT OR BREASTFEEDING MAY BE HARMFUL,” may be overlooked or not taken seriously by budtenders. Findings underscore the need for educational interventions aligned with California Senate Bill 540 (effective March 1, 2025, after our calls were completed) which requires that all California licensed cannabis retailers prominently display a brochure on the health risks of cannabis, including during pregnancy, at the point of sale or upon delivery and offer a printed copy to every new customer during their first purchase.^47^ This policy has potential to improve knowledge and awareness of risks of prenatal use for both budtenders and individuals of reproductive age. Future research is needed to test the implementation and impact of this policy on changes in budtender and pregnant individual awareness of potential harms and prenatal use. Policy enforcement, training opportunities, and national guidance related to product messaging and consumer protections may be useful ways to improve oversight and standardization of budtender recommendations.

Younger women, Black and Hispanic women, and those with lower socioeconomic status have higher rates of prepregnancy and prenatal cannabis use, more frequent cannabis use, and greater tobacco use.^4,30,40,48,49^ There are also existing inequities in access to high-quality health information. Future research is needed to assess whether policy changes, including implementation of more visible warnings around prenatal cannabis use, retailer training, and education reduce disparities in use.

### Strengths and Limitations

Strengths of this study include a novel, telephone-based randomized mystery caller survey study across California, with a robust sample, an adequate response rate, and use of scripts with and without mental health indications for use. Furthermore, this is the first study we know of to assess budtender recommendations about prenatal blunt use and the relative safety of different modes of prenatal cannabis use and to test whether responses varied by availability of delivery. Finally, the study took place during the seventh year of legal adult-use sales in California when most retailers were well-established, and we contextualized our findings using budtender quotes.

This study also had limitations. Calls were limited to storefront retailers with a California license to sell medical and adult-use cannabis and did not include retailers from others states or those operating without a retail license. We were not able to reach all retailers, primarily due to inactive or incorrect phone numbers, and results may not generalize to all licensed retailers if unreachable stores differed systematically from those contacted. Budtender recommendations may vary depending on the caller and who answered the phone, and single call snapshots may miss intrastore variability across staff and shifts. We did not collect budtender sociodemographics, and we were unable to assess whether advice varied with these factors. Calls were conducted over an 11-month period, during which social norms and the cannabis retail landscape may have changed, potentially influencing budtender perceptions. Finally, causal inferences about the effects of budtender advice on pregnant individuals’ behavior cannot be made in this cross-sectional study.

## Conclusions

This cross-sectional study was the first to examine budtender recommendations about prenatal blunt use. While most budtenders advised against prenatal blunt or tobacco use, 1 in 5 endorsed prenatal cannabis use as safe, highlighting the need for standardized budtender education about the health risks of prenatal use. Findings underscore the importance of accurate public policy information for pregnant individuals given that budtender advice may not align with medical guidelines or evidence-based research.

## Notes

Supplement 1. eFigure. Flowchart eTable. Cannabis Retailer Responses to Questions About Blunt, Tobacco, and Cannabis Use During Pregnancy, by Delivery Status

Supplement 2. Data Sharing Statement
