# Supplementary material for: Cannabis Retailer Advice on Blunt, Tobacco, and Cannabis Use During Pregnancy
Source: JAMA Netw Open. 2025 Dec 10;8(12):e2548373. doi: 10.1001/jamanetworkopen.2025.48373 (PMC13372022; doi:10.1001/jamanetworkopen.2025.48373)
Supplement: Supplement 1. — eFigure. Flowchart eTable. Cannabis Retailer Responses to Questions About Blunt, Tobacco, and Cannabis Use During Pregnancy, by Delivery Status [file jamanetwopen-e2548373-s001.pdf]

Supplemental Online Content

Young-Wolff KC, Does MB, Negusse R, et al. Cannabis Retailer Advice on Blunt, Tobacco, and Cannabis Use During Pregnancy. *JAMA Netw Open*. 2025;8(12):e2548373. doi:10.1001/jamanetworkopen.2025.48373

eFigure. Flowchart

eTable. Cannabis Retailer Responses to Questions About Blunt, Tobacco, and Cannabis Use During Pregnancy by Delivery Status

This supplemental material has been provided by the authors to give readers additional information about their work.

© 2025 Young-Wolff KC et al. *JAMA Network Open*.

eFigure. Flowchart

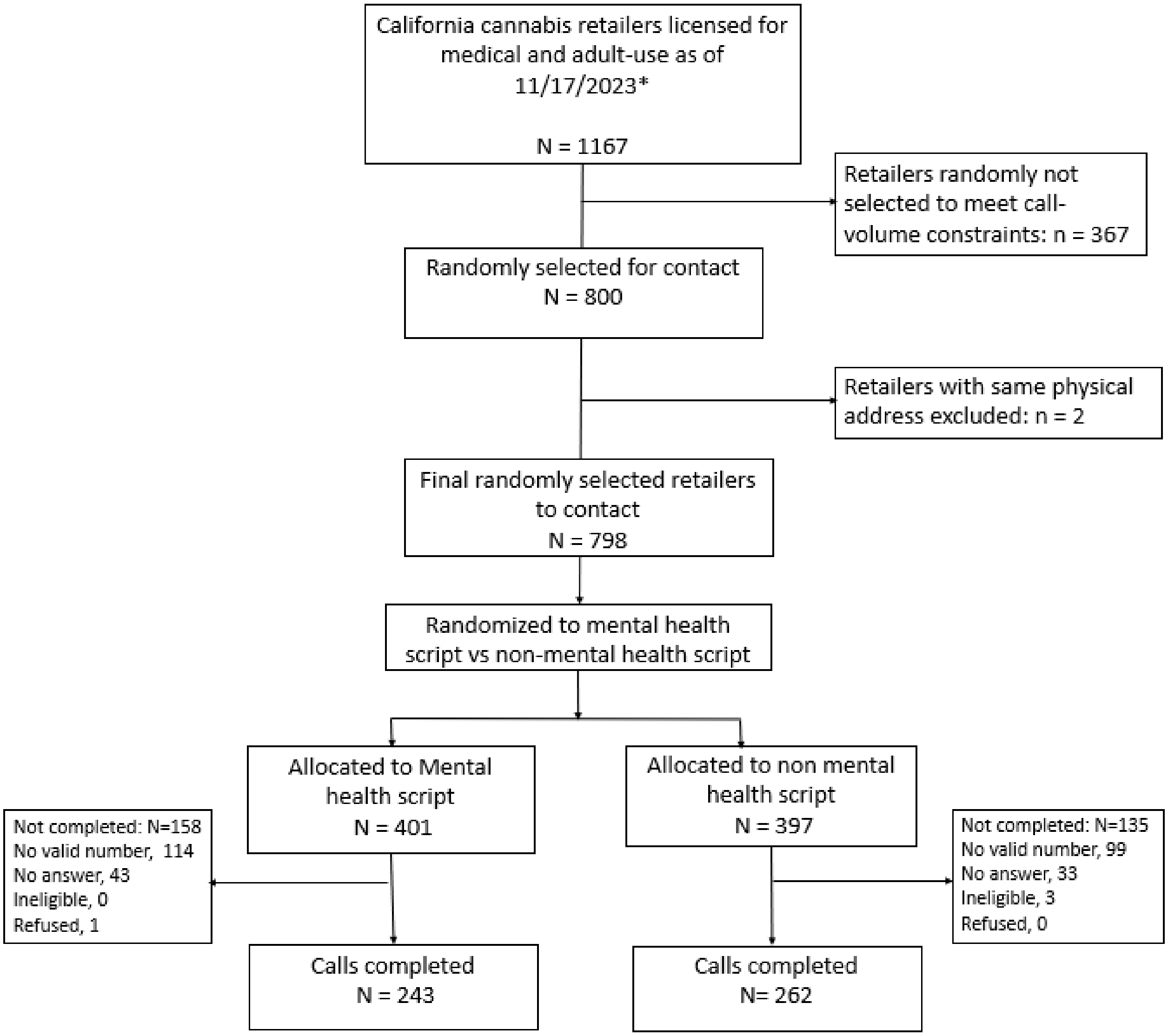

*Includes active storefront retailer licenses and microbusiness licenses endorsed to conduct adult-use and medical storefront retail activities in California as of 11/17/2023 from CA Department of Cannabis Control (DCC); excludes licensees with a medical only or adult-use only license designation. Calls were conducted over an 11-month period.

© 2025 Young-Wolff KC et al. *JAMA Network Open*.

eTable. Cannabis Retailer Responses to Questions About Blunt, Tobacco, and Cannabis Use During Pregnancy by Delivery Status

	Delivery Status						
	Overall N=505		Delivery N=166		No Delivery N=339		P-Value
Characteristic	N	% (95%CI)	N	% (95%CI)	N	% (95%CI)	
Prenatal Blunt Use[a]							0.97
Safe	4	0.8 (0.2-2.9)	1	0.6 (0.1-5.8)	3	0.9 (0.2-3.9)	
Not safe	402	79.6 (74.2-84.2)	134	80.7 (70.9-87.8)	268	79.1 (72.3-84.5)	
Unsure	15	3.0 (1.5-5.9)	5	3.0 (0.9-9.3)	10	2.9 (1.3-6.8)	
Cannot give medical advice	84	16.6 (12.5-21.8)	26	15.7 (9.3-25.1)	58	17.1 (12.1-23.6)	
Prenatal Tobacco Use[a]							0.94
Safe	4	0.8 (0.2-2.9)	1	0.6 (0.1-5.6)	3	0.9 (0.2-3.8)	
Not safe	400	79.2 (73.7-83.8)	133	80.1 (70.2-86.3)	267	78.8 (71.9-84.3)	
Unsure	29	5.7 (3.5-9.4)	8	4.8 (1.9-11.8)	21	6.2 (3.4-10.9)	
Cannot give medical advice	72	14.3 (10.5-19.2)	24	14.5 (8.4-23.7)	48	14.2 (9.7-20.3)	
Prenatal Cannabis Use[a]							0.21
Safe	104	20.6 (16.0-26.1)	26	15.7 (9.3-25.1)	78	23.0 (17.3-30.0)	
Not safe	204	40.4 (34.5-46.6)	71	42.8 (32.6-53.6)	133	39.2 (32.1-46.8)	
Unsure	97	19.2 (14.8-24.6)	31	18.7 (11.7-28.5)	66	19.5 (14.2-26.1)	
Cannot give medical advice	100	19.8 (15.3-25.2)	38	22.9 (15.1-33.1)	62	18.3 (13.2-24.9)	
Recommendations for Prenatal Use[b]							
Low/No THC[c]	185	36.6 (32.6-40.9)	63	38.0 (30.9-45.5)	122	36.0 (31.1-41.2)	0.67
Other harm reduction strategies[d]	111	22.0 (18.6-25.8)	36	21.7 (16.1-28.6)	75	22.1 (18.0-26.8)	0.91
Non-cannabis (e.g., mindfulness) [e]	31	6.1 (4.4-8.6)	13	7.8 (4.6-12.9)	18	5.3 (3.4-8.2)	0.27
No recommendations	239	47.3 (43.0-51.7)	64	38.6 (31.5-46.1)	129	38.1 (33.0-43.3)	0.91
Are certain modes safer than others[a]							0.84
Certain modes safer	211	41.8 (35.2-48.6)	72	43.4 (32.2-55.3)	139	41.0 (33.1-49.4)	
No difference in safety by mode	35	6.9 (4.2-11.3)	11	6.6 (2.7-15.2)	24	7.1 (3.9-12.6)	
No mode is safe	70	13.9 (9.8-19.3)	19	11.4 (5.8-21.2)	51	15.0 (10.0-22.0)	
Don't know	121	24.0 (18.6-30.3)	40	24.1 (15.5-35.5)	81	23.9 (17.5-31.7)	
Didn't ask	68	13.5 (9.5-18.8)	24	14.5 (8.0-24.8)	44	13.0 (8.4-19.6)	
Modes that are safer[b]							
Smoking[f]	28	5.5 (3.9-7.9)	12	7.2 (4.2-12.2)	16	4.7 (2.9-7.5)	0.25
Vaping[g]	11	2.2 (1.2-3.9)	6	3.6 (1.7-7.8)	5	1.5 (0.6-3.4)	0.12
Dabbing[h]	4	0.8 (0.3-2.0)	0	0.0 (0.0-2.3)	4	1.2 (0.5-3.0)	0.31
Edibles[i]	160	31.7 (27.8-35.9)	52	31.3 (24.8-38.7)	108	31.9 (27.1-37.0)	0.90
Sublingual[j]	5	1.0 (0.4-2.3)	2	1.2 (0.3-4.3)	3	0.9 (0.3-2.6)	0.67
Topical[k]	26	5.2 (3.5-7.4)	8	4.8 (2.5-9.2)	18	5.3 (3.4-8.2)	0.81
Modes that are less safe[b]							

© 2025 Young-Wolff KC et al. *JAMA Network Open*.

	Delivery Status						
	Overall N=505		Delivery N=166		No Delivery N=339		P-Value
Characteristic	N	% (95%CI)	N	% (95%CI)	N	% (95%CI)	
Smoking[f]	128	25.4 (21.8-29.3)	47	28.3 (22.0-35.6)	81	23.9 (19.7-28.7)	0.28
Vaping[g]	31	6.1 (4.4-8.6)	11	6.6 (3.7-11.5)	20	5.9 (3.9-8.9)	0.75
Dabbing[h]	5	1.0 (0.4-2.3)	2	1.2 (0.3-4.3)	3	0.9 (0.3-2.6)	0.67
Ingesting[i]	24	4.8 (3.2-7.0)	10	6.4 (3.5-11.4)	14	4.1 (2.5-6.8)	0.35
Edibles[j]	1	0.2 (0.0-1.1)	0	0.0 (0.0-2.3)	1	0.3 (0.1-1.7)	>.99
Topical[k]	0		0		0		---
Worth talking to doctor[a]							0.68
Yes (already suggested)	222	44.0 (37.3-50.8)	75	45.2 (33.9-57.0)	147	43.4 (35.4-51.7)	
Yes (when prompted)	233	46.1 (39.4-53.0)	79	47.6 (36.1-59.3)	154	45.4 (37.3-53.8)	
No	11	2.2 (0.9-5.2)	3	1.8 (0.4-8.5)	8	2.4 (0.8-6.5)	
I don't know	29	5.7 (3.3-9.8)	6	3.6 (1.1-11.1)	23	6.8 (3.6-12.3)	
Didn't ask	10	2.0 (0.8-5.0)	3	1.8 (0.-8.5)	7	2.1 (0.7-7.1)	
Source of information[b, l]							
No source	228	45.2 (40.9-49.5)	77	46.4 (39.0-54.0)	151	44.5 (39.3-49.9)	0.70
Personal experience/opinion	180	35.6 (31.6-39.9)	54	32.5 (25.9-40.0)	126	37.2 (32.2-42.4)	0.31
General knowledge	46	9.1 (6.9-11.9)	17	10.2 (6.5-15.8)	29	8.6 (6.0-12.0)	0.54
Published research	35	6.9 (5.0-9.5)	13	7.8 (4.6-12.9)	22	6.5 (4.3-9.6)	0.58
Online sources (e.g., Google)	40	7.9 (5.9-10.6)	16	9.6 (6.0-15.1)	24	7.1 (4.8-10.3)	0.32
Product/retailer warnings	29	5.7 (4.0-8.1)	10	6.0 (3.3-10.7)	19	5.6 (3.6-8.6)	0.85
Lack of reliable information	22	4.4 (2.9-6.5)	9	5.4 (2.9-10.0)	13	3.8 (2.3-6.4)	0.41
Script[a]							0.08
Mental health indication	243	48.1 (43.8-52.5)	89	53.6 (46.0-61.0)	154	45.4 (40.2-50.7)	
Non-mental health indication	262	51.9 (47.5-56.2)	77	46.4 (39.0-54.0)	185	54.6 (49.3-59.8)	

[a] Categories are mutually exclusive.
[b] Categories are not mutually exclusive.
[c] Products with no THC (e.g., products with only non-THC cannabinoids such as CBD, CBN, etc.) and low THC products (e.g., products noted as containing a THC:non-THC ratio, or a lower-than-average THC content) were combined into one category called low/no-THC.
[d] Harm reduction strategies include suggestions to use alternate (safer) methods of consuming that may still contain cannabis (e.g., using 'clean' methods like bong or pipe, switching to hemp blunt wraps, avoiding chemicals and pesticides) and suggestions to simply consume less cannabis.
[e] Non-cannabis recommendations include supplements, and lifestyle modifications such as diet, mindfulness, yoga, and meditation.
[f] Smoking includes the use of joints, bongs, pipes and blunts.
[g] Vaping is use of an electronic device that heats liquid or dry material to produce an aerosol or vapor (e.g., vape pens, desktop vaporizers).
[h] Dabbing is the use of cannabis concentrates (e.g., shatter, wax).
[i] Edibles includes gummies, tablets, capsules, tinctures, drinks, foods, and other orally ingested products.
[j] Sublingual are tablets or strips that dissolve under the tongue.
[k] Topicals are cannabis products applied directly to and absorbed through the skin (e.g., lotion, ointment, bath bombs).
[l] Source of information was recorded after the call based on the caller's assessment of the information provided. If budtender stated an obvious personal opinion (e.g., *I believe…*, *In my opinion…*), response was coded as personal opinion. If budtender cited specific research studies or specific websites, response was coded published research or online sources, respectively. If budtender

© 2025 Young-Wolff KC et al. *JAMA Network Open*.

	Delivery Status						
	Overall<br>N=505		Delivery<br>N=166		No Delivery<br>N=339		P-Value
Characteristic	N	% (95%CI)	N	% (95%CI)	N	% (95%CI)	
referenced specific retailer policies (e.g., this store prohibits giving medical advice) or warnings on products or posters displayed in dispensary, responses were coded accordingly. If responses reflected commonly held beliefs, (e.g., *most people say, it is generally not recommended*) without citing another source, responses were coded as general knowledge.							

© 2025 Young-Wolff KC et al. *JAMA Network Open*.
