# Supplementary material for: Cannabis Retailer Advice on Blunt, Tobacco, and Cannabis Use During Pregnancy
Source: JAMA Netw Open. 2025 Dec 10;8(12):e2548373. doi: 10.1001/jamanetworkopen.2025.48373 (PMC13372022; doi:10.1001/jamanetworkopen.2025.48373)
Supplement: Supplement 2. — Data Sharing Statement [file jamanetwopen-e2548373-s002.pdf]

Data Sharing Statement

Young-Wolff. Cannabis Retailer Advice on Blunt, Tobacco, and Cannabis Use During Pregnancy. *JAMA Netw Open*. Published December 10, 2025.
doi:10.1001/jamanetworkopen.2025.48373

Data
Data available: No
